# Giant ovarian yolk sac tumor during late pregnancy: a case report and literature review

**DOI:** 10.3389/fonc.2024.1437728

**Published:** 2024-09-06

**Authors:** Qin Wang, Jianxin Zuo, Chong Liu, Huansheng Zhou, Wenjie Wang, Yankui Wang

**Affiliations:** ^1^ Medical College, Qingdao University, Qingdao, China; ^2^ Department of Obstetrical, The Affiliated Hospital of Qingdao University, Qingdao, China; ^3^ Department of Gynecology, The Affiliated Hospital of Qingdao University, Qingdao, China

**Keywords:** late pregnancy, pregnancy with giant ovarian tumors, ovarian cancer, yolk sac tumor, malignancy during pregnancy

## Abstract

The manifestation of a giant ovarian yolk sac tumor during late pregnancy is relatively rare. A yolk sac tumor is a highly malignant germ cell tumor that originates from primitive germ cells. It is characterized by yolk sac differentiation *in vitro*. The frequency of prenatal examinations should be appropriately increased for ovarian tumors discovered during pregnancy. Furthermore, regular follow-up ultrasound should be performed, and tumor markers should be dynamically detected. If needed, imaging examinations such as computed tomography and magnetic resonance imaging should be combined to comprehensively investigate disease progression. If the tumor diameter and tumor marker levels rapidly increase during pregnancy, the possibility of malignancy increases. Therefore, exploratory laparotomy should be immediately performed to further improve subsequent treatment modalities, early diagnosis, early treatment, and prognosis. Herein, we report the case of a 28-year-old pregnant woman whose pregnancy was terminated at 29 weeks and 5 days. She complained of lower abdominal pain for 2 days. A pelvic mass was detected for 1 week, accompanied by increased levels of tumor markers such as serum alpha-fetoprotein, cancer antigen 125, carbohydrate antigen 724, and human epididymis protein 4. Imaging revealed the presence of a pelvic mass. At 32 weeks and 3 days of pregnancy, a cesarean section was performed, with a transverse incision in the lower uterine segment. Furthermore, pelvic adhesiolysis, omentectomy, right adnexectomy, right pelvic lymph node dissection, and pelvic metastasis peritonectomy were performed. The postoperative pathological diagnosis was yolk sac tumors of the ovary (stage IIB). Postoperatively, a five-cycle chemotherapy regimen comprising bleomycin, etoposide, and cisplatin was administered. During postoperative follow-up, the patient’s general condition was noted to be good, with the newborn and pregnant women ultimately achieving good outcomes. We reviewed the relevant literature to increase clinical doctors’ understanding of ovarian malignancy during pregnancy, guide treatment selection, and facilitate early intervention for associated diseases.

## 1 Case description

**Figure 1:**
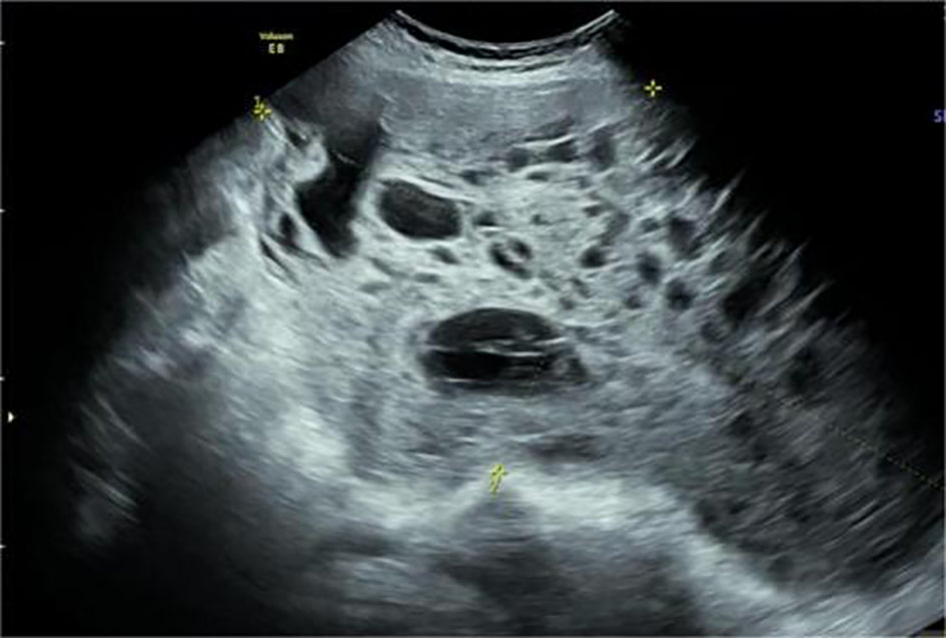
A solid cystic mass was detected in the right posterior lower part of the uterus. It was 18.43 × 8.47 × 10.53 cm in size. More compartments were observed in the cystic components, showing a “grid” shape.

**Figure 2:**
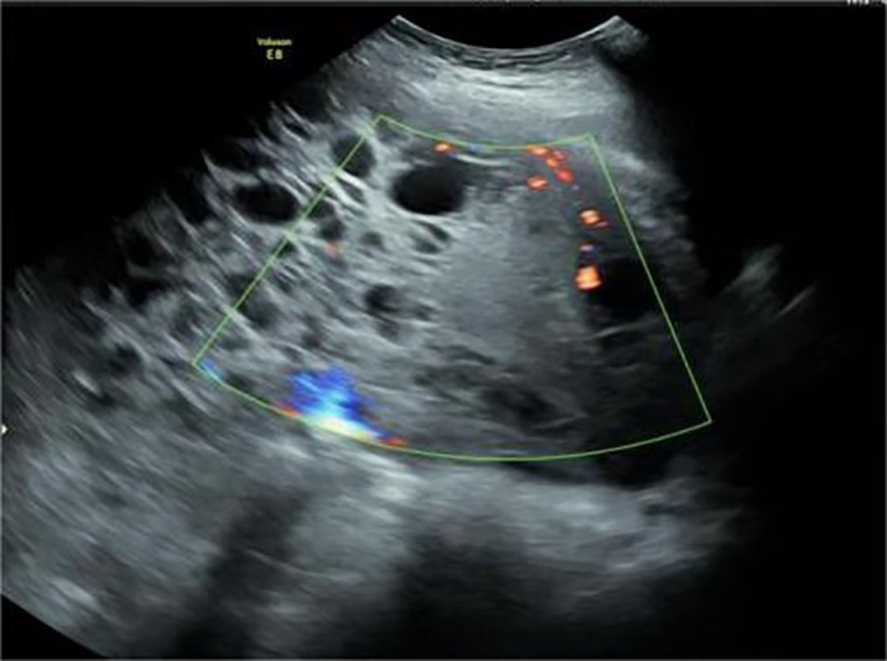
Uneven thickness separation and solid mass were observed in the cystic solid mass. Point stripe blood flow signals were observed in the separated and solid components.

The patient Yi was a 28-year-old woman with a regular menstrual cycle. Her last menstrual period was May 2, 2023. After 28 weeks and 5 days of gestation, an ultrasound at an external hospital revealed the presence of a cystic solid mass in the right posterior pelvic cavity of the uterus. The mass was approximately 14.7 × 10 × 8.6 cm in size. The solid ultrasound characteristics were primarily observed, with relatively uniform low echo and blood flow signals visible inside. The cystic part exhibited multiple liquid dark areas of varying sizes. The largest size range of these areas was approximately 5 × 1.9 cm, with good internal translucency. The liquid dark areas were observed in the abdominal cavity, peri liver, and right abdomen. The deepest depth of the liquid was approximately 4.4 cm in the right abdomen, with good internal translucency. Preoperatively, the blood cancer antigen 125 (CA125) level was 260.9 U/mL. Untreated. Two days before admission, the patient experienced lower abdominal pain. She was admitted to our hospital on November 26, 2023. Ultrasound revealed the presence of a cystic solid mass sized 18.8 × 10.8 × 9.2 cm in the right posterior part of the uterus. The mass had a regular shape and clear boundaries. The edge of the mass exhibited rich blood flow signals (**Figures 1**, **2**). Pelvic magnetic resonance imaging (MRI) revealed the presence of a cystic solid mass in the right posterior part of the uterus, presenting as an equal-length T1 mixed with long and short T2 signal shadows. Diffusion-weighted imaging (DWI) revealed mixed signals, with a size of approximately 95 mm × 148 mm, and a close association with the uterus and adnexa. The tumor markers levels were as follows: CA125: 471.9 U/mL, human epididymis protein 4: 62.6 pmol/L, and alpha-fetoprotein (AFP): 41790 ng/mL. The blood routine findings were as follows: white blood cell (WBC): 16.11 × 10^9^/L and hemoglobin (Hb): 123 g/L. Blood biochemistry findings were as follows: albumin: 37.8 g/L. At admission, the patient (G1P0) was initially diagnosed with threatened preterm labor, pregnancy complicated with a pelvic tumor, and pregnancy diabetes. Magnesium sulfate was administered to nourish the fetal brain nerves, and dexamethasone was administered to promote fetal lung maturation. Subsequent blood routine examination revealed the following: WBC: 17.43 × 10^9^/L, Hb: 96 g/L, CRP: 50.27 mg/L, PCT: 0.223ng/mL, and albumin: 25.9 g/L. The patient received symptomatic treatment such as “piperacillin sodium tazobactam” anti-infection and albumin infusion.

**Figure 3:**
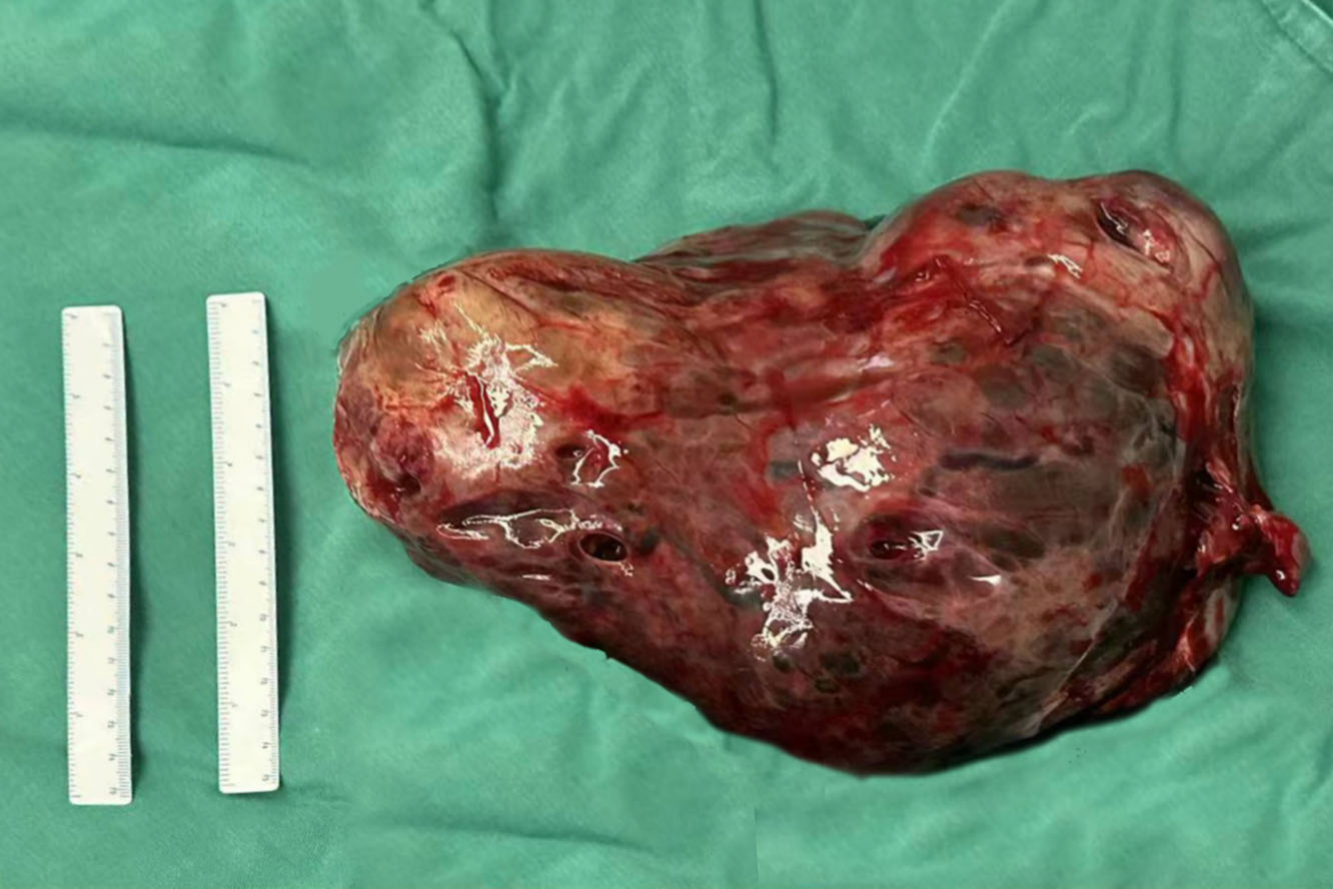
The tumor was completely excised. It was approximately 25 × 15 × 15 cm in size.

**Figure 4:**
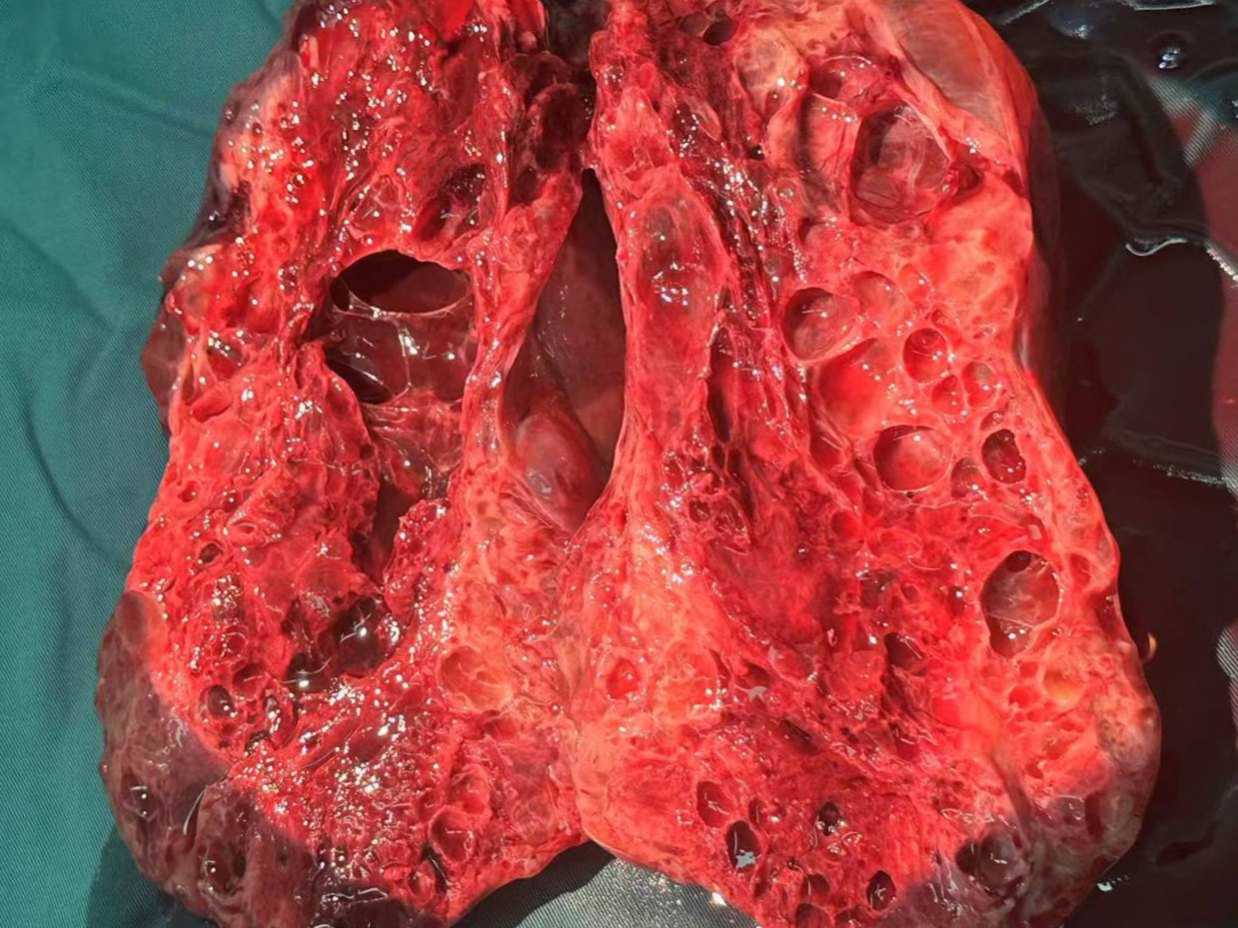
Internal view of the dissected tumor.

At 32 weeks of gestation, a follow-up ultrasound revealed a significant increase in tumor size compared with the previous observation, with an increase in AFP levels. After multidisciplinary consultation, we decided to terminate the pregnancy and administered meropenem as an anti-infective treatment. On December 15, 2023, a transverse incision cesarean section was performed in the lower segment of the uterus. A male infant was delivered, with an Apgar score of 8 points at 1 min and a weight of 1960 g. The infant was transferred to the neonatology department. During the operation, we observed the enlargement of the right ovary, with a size of approximately 25 × 15 × 15 cm. The ovary was extremely fragile but without an evident capsule. **Figures 3**, **4** illustrate the gross specimen. No remarkable anomalies were observed in the appearance of the right fallopian tube, and the right adnexa adhered to the lower segment of the posterior uterine wall, bilateral posterior lobes of the broad ligament, anterior rectum, and uterine rectal depression peritoneum. The right posterior lobe of the broad ligament peritoneum was noted to be thickened. After blunt separation and adhesion, extensive bleeding was observed on the wound. The appearance of the left attachment was not abnormal; however, and no evident metastatic nodules were observed in the omentum, liver, spleen, diaphragm, and abdominal intestinal tract. The lymph nodes adjacent to the abdominal aorta and pelvic cavity were not palpable and enlarged. Right adnexectomy was performed, and frozen section analysis during the operation resulted in the diagnosis of a yolk sac tumor. The patient’s family was communicated, and they requested staged surgery to preserve fertility. Accordingly, we decided to perform omentectomy, right pelvic lymph node dissection, and pelvic metastasis peritonectomy. Postoperative paraffin pathology revealed the following: (right) a malignant ovarian tumor with extensive bleeding and necrosis, combined with morphological and immunohistochemical analysis results, consistent with a yolk sac tumor (size 17.5 × 16 × 7 cm). However, no tumor was observed in the tubal tissue. Immunohistochemical analysis revealed the following: SALL4 (+), OCT4 (weak+), AFP (+), GPC3 (+), CD30 (−), Pax-8 (−), NapsinA (−), ER (−), PR (−), WT-1 (−), p53 (+, approximately 20%), Vimentin (−), CKpan (+), EMA (-), Ki-67 (+, approximately 60%). Ascites smear and examination revealed the absence of malignant tumor cells in the lymph nodes.

The patient was discharged from the hospital on day 9 postoperatively. On the day of discharge, AFP levels were 3650 ng/mL. On day 19 postoperatively, a chemotherapy regimen comprising bleomycin + etoposide + cisplatin (BEP) was administered. After the patient completed five chemotherapy cycles, her condition was generally good, with a significant decrease in tumor markers and reaching the normal range. The tumor marker levels on April 2, 2024 were as follows: AFP: 6.48 ng/mL and CA125: 9.5 U/mL. **Tables 1**, **2** present the fluctuation curves of the tumor markers.

**Table 1:** Fluctuation curves of AFP.

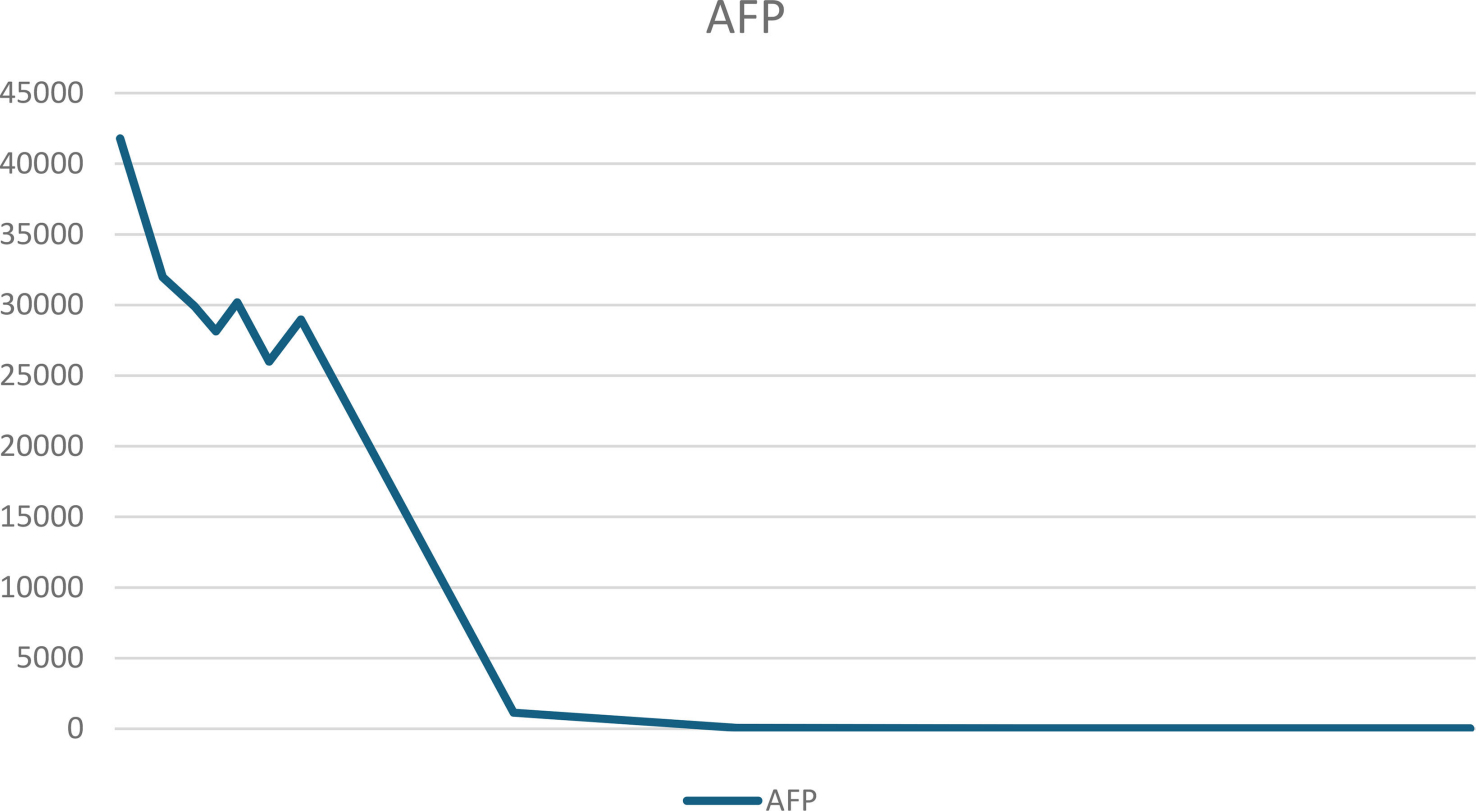

**Table 2:** Fluctuation curves of CA125.

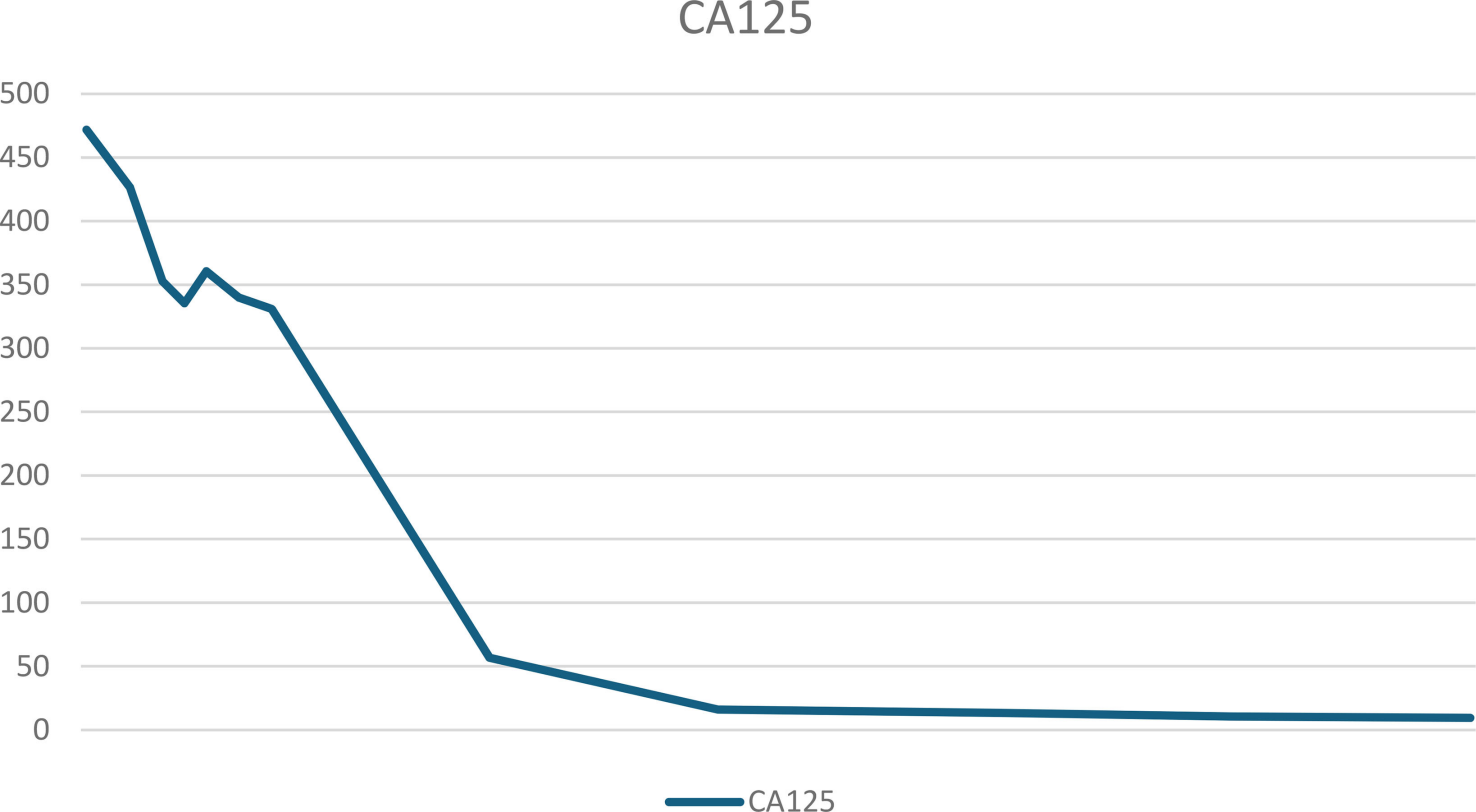

## 2 Discussion

### 2.1 Clinical manifestations and pathological features

Ovarian yolk sac tumor (OYST), also called ovarian endoembryonic sinus tumor, is a type of ovarian germ cell malignant tumor (MOGCT) derived from primordium germ cells. MOGCT currently lacks specific clinical predictors. Classic serum tumor markers include AFP, β-HCG, anti-Miller tube hormone (AMH), CA125, and CA199, but most of which are interfered by pregnancy status. The clinical prognosis of ovarian germ cell malignancy (MOGCT) is poor, and the adverse factors include age >45 years, pathological stage > I, incomplete surgical resection and yolk sac tumor (YST) histology (1). This tumor type is characterized by yolk sac differentiation *in vitro* and accounts for approximately 5% of ovarian malignancies. OYST may occur in children and women of childbearing age, with a poor prognosis (2, 3). The tumor tissue is mostly an oval nodular or a lobulated mass, with an envelope, soft substances, cystic solids, and grayish-yellow to colorful sections and accompanied by bleeding and necrosis.

**Figure 5:**
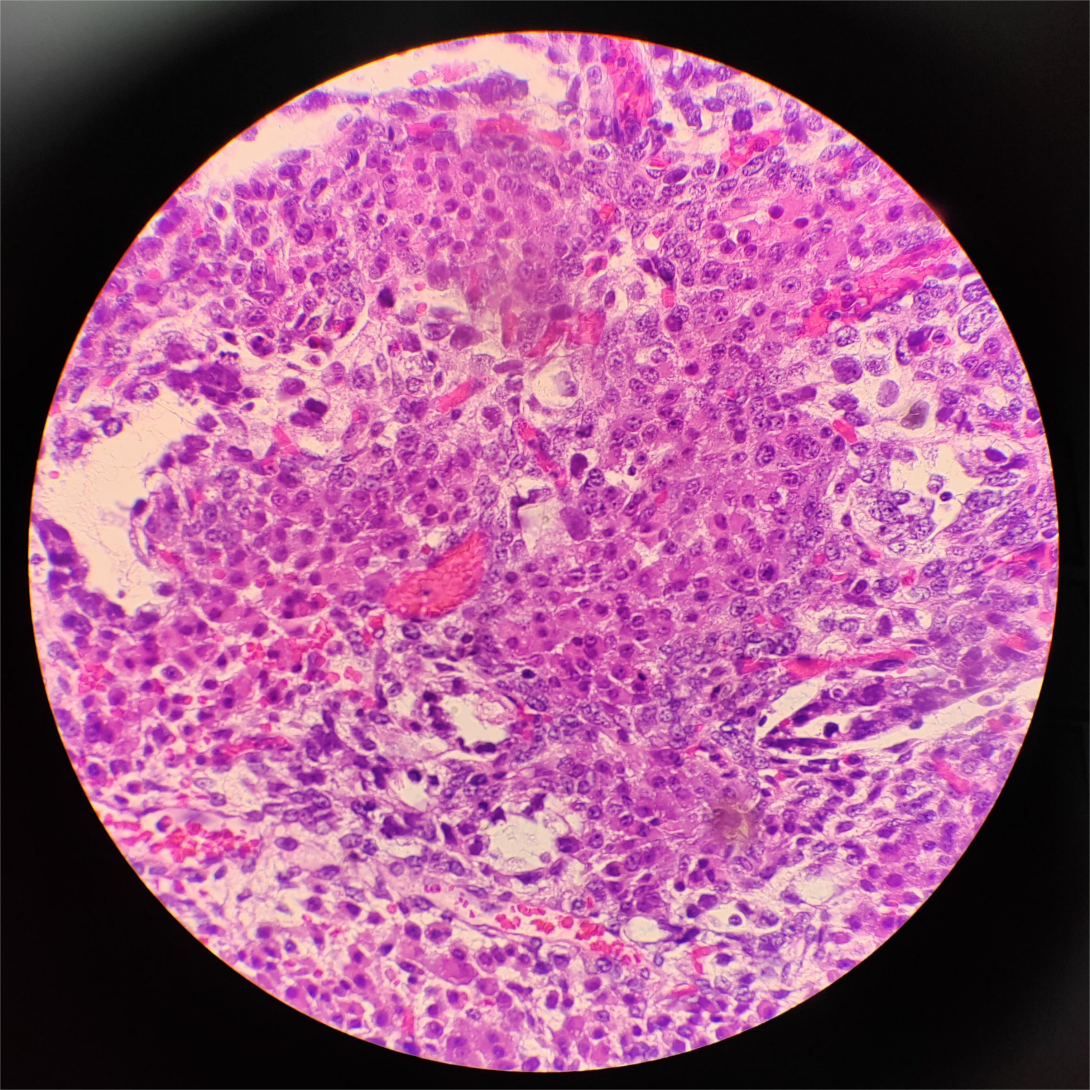
Hematoxylin and eosin staining of the yolk sac tumor. Solid areas present within the tumor in this patient, ×40.

**Figure 6:**
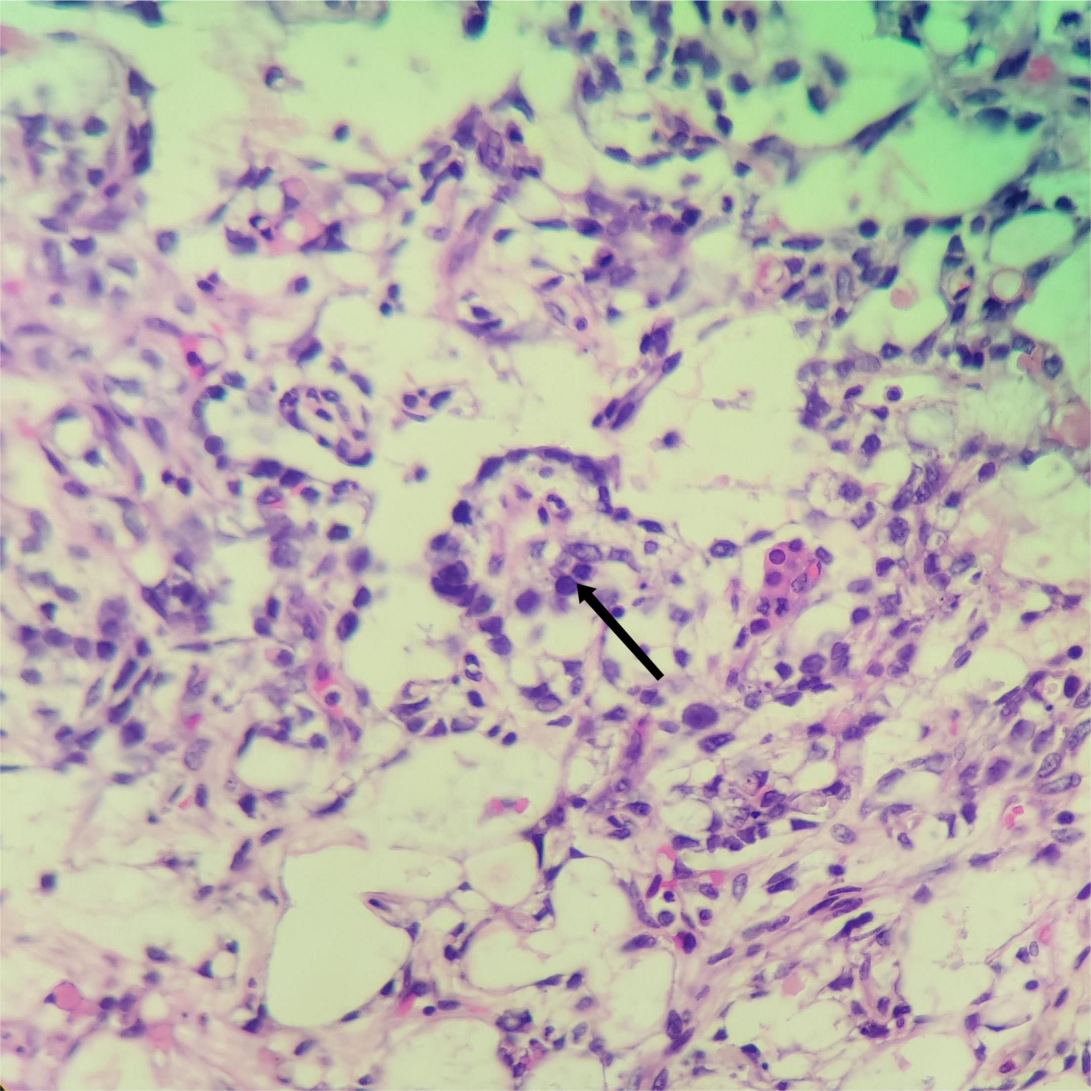
Hematoxylin and eosin staining of the yolk sac tumor. Black arrows indicate the Schiller–Duval) corpuscles in this tumor, ×200.

The pathological manifestation is the differentiation of various endoderms and their derivatives. The reticular/microcapsule type is the most common pathological manifestation observed under a microscope. The endoderm sinus type (Schiller–Duval corpuscles) and glandular type (endometrioid type) are some other pathological morphologies (4). Tumor-specific Schiller–Duval bodies with diagnostic significance are observed in the papillary region, which is characterized by papillary structure formation by tumor cells around the axis of the fiber vessels in the cystic cavity (5, 6) (**Figures 5**, **6**). AFP is 100% expressed in pure OYST. Furthermore, it can be expressed in mixed tumors with yolk sac tumor components (2, 7). Glypican-3, which sensitivity is 3 times higher than AFP for OYST, but less specific. And other immunohistochemical markers that are also typically positive in OYST include SALL4、 Lin28、 CDX2、 NANOG、 PLAP、 CD30、 HepPar1、 CD117、 OCT3/4 and so on. Furthermore, SALL4 is useful in distinguishing OYST from ovarian clear cell adenocarcinoma (2, 8). In the present case, characteristic Schiller–Duval bodies were observed during pathological examination, as indicated by an arrow in **Figure 6**.

Most patients with OYST present with chronic pelvic and abdominal pain as the primary initial symptom. MOGCT rapidly grows, and most patients have corresponding clinical symptoms in the early disease stage. Approximately 70% of patients with MGOCT present with abdominal pain and abdominal mass as the primary symptoms, followed by abdominal distension (15%), fever (10%), and abnormal vaginal bleeding (5%–10%), with some patients exhibiting irregular menstruation (4). Acute abdominal manifestations such as intracystic hemorrhage, torsion, and rupture are observed in 10% of patients with OYST. Furthermore, approximately 10% of patients with OYST present with an asymptomatic pelvic mass, with irregular vaginal bleeding and infertility also being important symptoms (4). However, owing to the particularity of pregnancy, lower abdominal pain is the primary clinical symptom of pregnancy complicated with OYST, and some symptoms are masked by the pregnancy status, making pregnancy complicated with OYST diagnosis more challenging. In the present case, abdominal pain was the initial symptom, and the pelvic mass diameter rapidly increased without evident directional characteristics.

### 2.2 Auxiliary examination

No specific tumor markers are available for OYST during pregnancy and pregnancy interferes with most relevant tumor markers. Fetal antigens such as AFP, beta-human chorionic gonadotropin (β-HCG), and CA125 are detected during pregnancy. Furthermore, the levels of various tumor markers physiologically increase during pregnancy and fluctuate with changes in gestational age. Therefore, the diagnostic value of commonly used tumor markers is limited during pregnancy. Some researchers have proposed that continuous monitoring during pregnancy may facilitate differential diagnosis (9). The AFP levels in our patient were significantly higher than the acceptable range during pregnancy, with the highest level of 41790 ng/mL. Furthermore, during dynamic monitoring, the tumor markers were maintained at high levels in our patient.

Ultrasound examination is a common method for evaluating OYST during pregnancy. The internal echoes of OYST exhibit various manifestations, including solid, cystic, or mixed cystic and solid, particularly the last one. When the tumor is extremely large, cystic changes with varying degrees of hemorrhage and necrosis may occur, with the potential differentiation of other tissue components in the lesion. In general, the solid part of OYST exhibits rich blood flow signals, with medium and low blood flow resistance (10). However, with an increase in the gestational age, the uterus gradually enlarges, with ultrasound evaluation gradually becoming difficult. MRI can be more helpful for determining tumor nature, identifying the degree of the malignant tendency of the tumor, and evaluating the relationship between the tumor and surrounding tissues to help and make treatment decisions. MRI plain scan revealed that the lesions were primarily isointense and low signal on T1WI but isointense and high mixed signal on T2WI. The cystic necrosis area exhibited longer T1 and T2 signals. Furthermore, contrast-enhanced MRI revealed uneven mass enhancement, tortuous blood vessels, no enhancement in the cystic necrosis area, and an expanded enhancement range in the delayed phase, with gradual enhancement (11, 12). However, some studies have revealed that the MRI contrast agent can penetrate the placenta and be excreted by the fetal kidneys into the amniotic fluid, which can easily affect the imaging interpretation (13). Moreover, animal experiments have verified that contrast media can increase the incidence of skeletal malformations in offspring; therefore, caution should be exercised when performing enhanced MRI during pregnancy (9). In this case, B-ultrasound revealed the presence of a predominantly solid cystic mass with a clear boundary, uneven septa, and solid mass within. Furthermore, dotted and strip-like blood flow signals were observed in the septa and solid parts. Lastly, pelvic MRI revealed equal-length T1 mixed long and short T2 signal shadows, and DWI revealed mixed signals, which were closely associated with the uterus and adnexa.

### 2.3 Treatment and prognosis

In general, the incidence of pregnancy complicated with ovarian tumors is low, and pregnancy complicated with ovarian malignant tumors is even more rare. 90% of ovarian cancers are of an epithelial cell type and comprise multiple histologic types, with various specific molecular changes, clinical behaviors, and treatment outcomes. The remaining 10% are non-epithelial ovarian cancers, which include mainly germ cell tumors, sex cord-stromal tumors, and some extremely rare tumors such as small cell carcinomas. Germ cell tumors are the most common ovarian neoplasms in women until 30 years of age and most of the patients are diagnosed with early-stage disease (60–70%) (14). Some researchers have reported that pregnant women have abundant blood supply in the pelvis; increased blood estrogen, progesterone, and β-HCG levels; and an altered immune system in the body. This promotes the proliferation of malignant tumor cells, which may infiltrate and metastasize, affecting the prognosis (15).

Owing to the particularity of pregnancy, comprehensively considering many factors during the clinical treatment of such diseases is essential. The pathological type, stage, fertility requirements, and expectations of this pregnancy as well as fetal factors should be considered to achieve a win–win situation. For patients with high suspicion of malignancy during early pregnancy, standard ovarian cancer treatment should be recommended after pregnancy termination (16). During early pregnancy, the ovarian corpus luteum plays a role in completing hormone secretion and maintaining the pregnancy. If surgery is performed in the early pregnancy stage, the risk of abortion will be significantly increased owing to damage to ovarian function. Patients with strong fertility requirements can be closely monitored until the second trimester of pregnancy for surgery (16). For those in the third trimester, pregnancy can be actively terminated and standardized surgery can be performed after completing fetal lung maturation promotion. Surgery is the primary treatment modality for OYST. Previously, the surgical principles of OYST referred to the surgical principles of ovarian epithelial tumors (2), and radical surgery was performed, i.e., laparotomy with total abdominal hysterectomy, bilateral salpingo-oophorectomy, pelvic lymphadenectomy, para-aortic lymphadenectomy, and ascites exfoliative cell examination. Because most patients with OYST have fertility requirements, the surgical modality that preserves reproductive function has become the basic treatment principle (17). Regardless of the tumor stage, the uterus and at least one ovary or a part of the ovarian tissue can be preserved (2, 17). Therefore, the surgical principle of OYST during pregnancy is similar to that during non-pregnancy. Tewari et al. (18) reported six cases of OYST during pregnancy, of which five cases underwent unilateral salpingo-oophorectomy during 10–26 weeks of pregnancy, whereas 1 of them received three cycles of the BEP chemotherapy regimen owing to metastatic dysgerminoma. All patients delivered their infants after 35 weeks, with no evident anomalies in the five newborns. The sixth patient, who was diagnosed with mixed germ cell tumor stage IIIC, opted for pregnancy termination at 18 weeks of gestation and immediately started adjuvant systemic chemotherapy. After 14 weeks of pregnancy, the placenta can secrete enough hormones to maintain pregnancy and decrease the risk of abortion, with enough free space for surgery in the pelvic cavity. At present, most researchers recommend 16–18 weeks of pregnancy as the best operation time (16). If emergency symptoms such as severe abdominal pain are present and tumor torsion or rupture is suspected, emergency surgery should be performed regardless of the gestational age. Most OYSTs are the cystic solid type inside, with most exhibiting different degrees of cystic changes such as hemorrhage and necrosis. Furthermore, most OYSTs have a large diameter, with a median diameter of approximately 20 cm (2). In addition, the capsule is fragile, prone to rupture, and difficult to remove after resection, restricting the application value of laparoscopy in this disease. In the present case, we detected a large pelvic mass in the third trimester of pregnancy. The tumor had a large diameter and rapidly grew. After completing fetal lung maturation promotion, a cesarean section was performed in the third trimester of pregnancy to terminate the pregnancy, followed by standardized surgery so that the newborn and pregnant women could achieve maximum benefits.

The current guidelines for the diagnosis and treatment of ovarian malignant tumors recommend postoperative chemotherapy for MOGCT, except for stage I dysgerminoma and stage I G1 immature teratoma (4). Furthermore, the current guidelines for OYST recommend BEP as the first-line chemotherapy regimen for MOGCT (17). Tumor marker monitoring should be given attention during chemotherapy. Furthermore, two cycles of consolidation chemotherapy should be administered after the tumor markers are within the normal range. At present, no definite conclusion is available regarding the effect of chemotherapy drugs on pregnancy and the fetus during chemotherapy for pregnancy complicated with OYST. Furthermore, large sample research data are lacking. Jorine de Haan et al. (19) collected 1170 cases of pregnancy complicated with malignant tumors and investigated the outcomes of pregnant patients, i.e., obstetric and neonatal outcomes. They reported the low risk of fetal malformations caused by chemotherapy in the second and third trimester of pregnancy; however, infants who received prenatal chemotherapy may exhibit an increased risk of complications compared with those who did not receive chemotherapy. For example, the incidence of low-birth-weight infant and neonatal intensive care unit admission increased. The 5–10-week gestation period is important for fetal organ differentiation, during which exposure to cytotoxic drugs increases the risk of teratogenesis (20). To prevent damage to fetal liver and kidney function and myelosuppression of the mother and newborn by chemotherapy drugs, chemotherapy should be stopped after 35 weeks of gestation and 3 weeks before delivery (9, 21). The gestational age of our patient was approximately 30 weeks when the tumor was detected, and the disease continued to progress during the observation. Considering that the gestational age had reached 32 weeks and the possibility of malignancy was high, we decided to terminate the pregnancy and performed a cesarean section to deliver a boy with a 1-min Apgar score of 8 and weight of 1960 g.

In summary, pregnancy complicated with OYST is a rare condition, and pregnancy status may obscure its specific clinical features; therefore, making a clear diagnosis is challenging. Furthermore, owing to physiological fluctuations in tumor marker levels during pregnancy, tumor markers have limited diagnostic value, warranting the dynamic and continuous monitoring of the changes in tumor markers and relying on auxiliary examinations such as ultrasound, CT, and MRI to aid diagnosis. OYST should be considered when pregnant women present with a large pelvic mass with abdominal pain, and ultrasound reveals predominantly cystic and solid echo with rich blood flow signals. If the tumor diameter continues to increase during pregnancy, clinicians should be vigilant and highly suspect malignancy. After assessing the gestational age and fetal condition, early intervention should be performed to achieve early detection, diagnosis, and treatment and improve prognosis.

## Data Availability

The datasets presented in this study can be found in online repositories. The names of the repository/repositories and accession number(s) can be found in the article/supplementary material.
